# Integrated Single-Cell and Bulk RNA-Sequencing Analysis Identifies an Aging-Related Gene Signature for Prognosis in Breast Cancer

**DOI:** 10.3390/genes17080921

**Published:** 2026-08-04

**Authors:** Pengcheng Chen, Yindan Lin, Jingjia Li, Yiwei Gu, Xueyun Zhang

**Affiliations:** 1 School of Artificial Intelligence, Taizhou University, Taizhou 318000, China; 2 Markey Cancer Center, University of Kentucky, Lexington, KY 40536, USA; 3 Department of Toxicology and Cancer Biology, College of Medicine, University of Kentucky, Lexington, KY 40536, USA; 4 Human Phenome Institute, Zhangjiang Fudan International Innovation Center, Fudan University, Shanghai 201203, China; 5 School of Medicine, Taizhou University, Taizhou 318000, China

**Keywords:** breast cancer (BRCA), aging- and senescence-induced genes (ASIGs), tumor microenvironment (TME), risk score model, single-cell RNA sequencing

## Abstract

Background: Cellular senescence exerts a complex influence on BRCA progression and TME remodeling. However, the specific roles of ASIGs in regulating the TME and determining patient outcomes remain unclear. Methods: Using TCGA (training), METABRIC (validation), and single-cell RNA-seq datasets, we systematically characterized ASIGs in BRCA. Prognostic ASIGs were identified to define molecular subtypes and construct a 17-gene LASSO-Cox risk model, which was integrated with clinical factors to develop a prognostic nomogram. Microenvironmental features and cell–cell communication networks were deconstructed using computational deconvolution and single-cell algorithms (SCISSOR and CellChat). Results: We established a robust 17-gene ASIG-based prognostic signature that effectively stratified BRCA patients into high- and low-risk groups and served as an independent prognostic predictor (HR = 3.94, *p* < 0.001). The nomogram accurately predicted 1-, 3-, and 5-year overall survival. Notably, the two risk groups exhibited strikingly distinct TME landscapes. The low-risk group was characterized by a coordinated, B cell-centric immune network, whereas the high-risk group displayed T cell exhaustion and immunosuppressive myeloid infiltration. Conclusions: The ASIG-based prognostic risk model is independent of traditional clinicopathological factors, providing a robust tool for patient risk stratification and offering biological insights into senescence-driven microenvironmental remodeling.

## 1. Introduction

Breast cancer (BRCA) is the most common malignancy and a leading cause of cancer death in women. In the United States alone, an estimated 313,570 new cases were projected for 2024 [1]. Clinical progress is consistently hampered by the disease’s extreme heterogeneity, making reliable diagnosis, prognosis, and therapeutic choices notoriously difficult [2,3]. Among the numerous factors influencing its pathogenesis and clinical behavior, aging has emerged as a pivotal determinant of tumor development, progression, and patient outcomes [4,5,6]. The increasing clinical attention given to both younger and elderly BRCA patients highlights the importance of exploring age-associated biological mechanisms that shape tumor evolution [7,8].

Cancer is a complex disease driven by genetic instability, abnormal epigenetic modifications, and dysregulated cellular proliferation that promote malignant invasion and metastasis. Over the past few decades, our understanding of cancer biology has rapidly advanced, shifting the paradigm of therapeutic management from traditional broad-spectrum approaches, such as surgery, radiotherapy, and cytotoxic chemotherapy, to highly precise modern modalities including targeted small-molecule inhibitors, antibody–drug conjugates (ADCs), and novel immunotherapies [9]. Despite these breakthroughs, clinical outcomes and therapeutic durability are frequently compromised by intrinsic and acquired drug resistance, intratumoral heterogeneity, and suppressive tumor microenvironments (TMEs). Therefore, dissecting the precise molecular mechanisms that govern tumor evolution and microenvironmental interactions remains essential for identifying novel therapeutic targets and refining patient stratification strategies.

However, the link between breast cancer and aging extends beyond chronological age to the complex process of biological aging, which fosters a microenvironment conducive to tumorigenesis. Biological aging is underpinned by interconnected hallmarks [10], such as cellular senescence, epigenetic alterations, and altered intercellular communication. Aging drives a cascade of molecular changes, from genomic instability to the buildup of somatic mutations [11,12]. A key consequence is the emergence of senescent cells, which permanently exit the cell cycle in response to damage [13,14]. However, this process is paradoxically double-edged. Senescent cells can promote tumor progression through the senescence-associated secretory phenotype (SASP) [15,16], a complex pro-inflammatory program involving cytokines, chemokines, growth factors, and proteases [17,18,19]. The SASP remodels the TME by promoting angiogenesis, fibrosis, and immune evasion, ultimately facilitating tumor growth and metastasis [20,21]. Due to this critical, context-dependent duality of senescence, it is imperative to identify molecular signatures capable of predicting its ultimate effect on BRCA prognosis [22,23].

Growing evidence links dysregulated senescence pathways to poor outcomes in BRCA [24,25] and other malignancies. Senescence can alter the immune landscape of tumors and foster an immunosuppressive environment [16]. For instance, chronic activation of SASP factors such as IL-6 [26], IL-8 [27,28], and TGF-β [29] promotes the recruitment of myeloid-derived suppressor cells and M2-like macrophages, while impairing cytotoxic T cell activity [26]. Consequently, the aging-associated TME tends to exhibit reduced lymphocyte infiltration and diminished antitumor immune surveillance [30,31]. However, a systematic map of how aging- and senescence-induced genes (ASIGs) collectively shape the TME and determine patient prognosis remains lacking.

Therefore, in the present study, we define aging-related genes from core biological pathways of aging and senescence, hypothesizing that transcriptional signatures reflecting their dysregulation within the TME serve as robust prognostic biomarkers independent of chronological age. Levering recent advances in high-throughput sequencing, we integrate bulk and single-cell transcriptomic datasets [32] to dissect these complex interactions. This integrative strategy allowed us to systematically characterize prognostic ASIGs, map the aging-associated TME landscape, and establish a robust risk model, thereby providing novel insights into the biological and clinical significance of aging-associated processes in breast cancer.

## 2. Materials and Methods

### 2.1. Data Collection

To establish a candidate gene set, we curated aging- and senescence-induced genes (ASIGs) by integrating seven curated gene sets from the Molecular Signatures Database (MSigDB, v7.4) [33] known to be upregulated in cellular senescence: GOBP Cell Aging (M14701), Tang Senescence Tp53 Targets Up (M11850), WP TCA Cycle in Senescence (M40058), WP Senescence and Autophagy in Cancer (M39619), GOBP Regulation of Cellular Senescence (M16568), GOBP Positive Regulation of Cellular Aging (M24705), and GOBP Replicative Senescence (M14683). These gene sets were selected based on their confirmed involvement in cellular aging and senescence processes [11,34].

Transcriptomic read counts, Transcripts Per Million (TPM) expression values, and associated clinical data were acquired from TCGA as the training set, covering a cohort of 1058 BRCA patients with primary solid tumor samples. For external validation, normalized, log_2_-transformed microarray-based gene expression data and clinical information for 1980 breast tumor samples were collected from the METABRIC database [35]. For these samples, we downloaded them through the cBioPortal platform (https://www.cbioportal.org/study/summary?id=brca_metabric, accessed on 5 March 2024). The single-cell RNA-sequencing (scRNA-seq) data used in this study were obtained from a public human breast cancer single-cell atlas, published by Wu et al. in Nature Genetics (2021) [36]. The dataset was accessed through the Broad Institute Single Cell Portal under the accession number SCP1039 [36]. Patients lacking complete overall survival (OS) or clinical follow-up data were excluded. Baseline patient characteristics are summarized in Supplementary Table S1.

### 2.2. Identification of Prognosis-Related ASIGs and Molecular Subtyping

**Figure 1:**
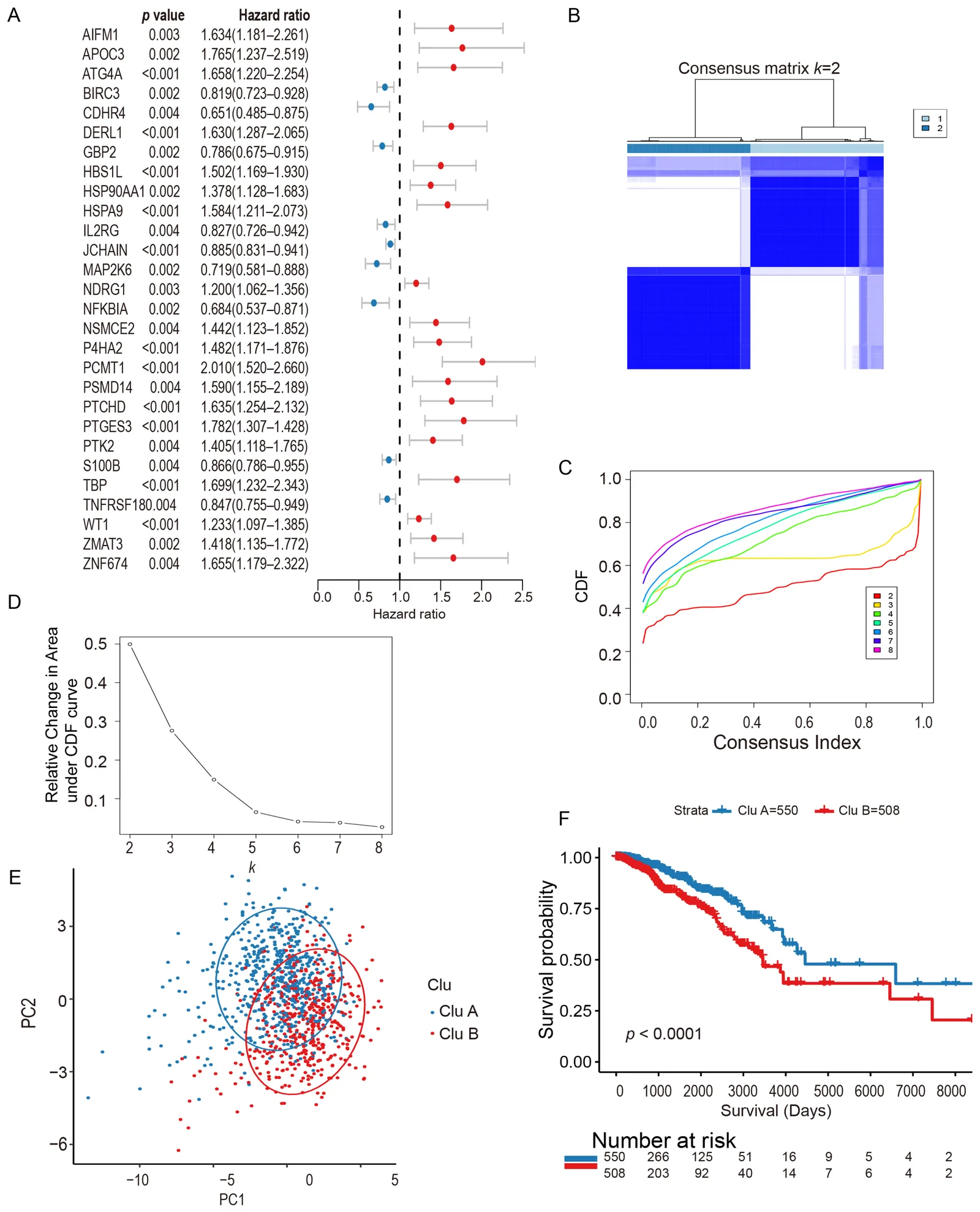
Identification of ASIG-based molecular subtypes with distinct prognoses in BRCA. (**A**) Forest plot of the univariate Cox regression analysis for prognostically significant ASIGs. Squares represent the hazard ratio (HR), and horizontal lines indicate the 95% confidence interval (CI). (**B**) Consensus clustering matrix of the TCGA-BRCA cohort for *k* = 2 based on the expression of prognostic ASIGs. (**C**) Cumulative Distribution Function (CDF) curves for *k* = 2 to *k* = 8. The shape of the CDF curve provides a quantitative measure of clustering stability. A flatter curve indicates more stable clusters. (**D**) Delta area plot illustrating the relative change in the area under the CDF curve for *k* = 2 to *k* = 8. (**E**) Principal component analysis (PCA) plot showing the distribution of the two distinct clusters. (**F**) Kaplan–Meier curves for OS of patients in Cluster A (*n* = 550) and Cluster B (*n* = 508). The *p*-value was calculated using the log-rank test. Number-at-risk tables are shown below.

Prognostic ASIGs were identified in the TCGA cohort using univariate Cox regression with OS as the primary endpoint. Unsupervised consensus clustering (ConsensusClusterPlus R package, version 1.56.0) was performed on the expression matrix of these prognostic ASIGs [37]. The optimal cluster number (*k* = 2) was evaluated using consensus matrices, Cumulative Distribution Function (CDF) curves, and delta area plots (Figure 1B–D, Supplementary Figure S1). Principal component analysis (PCA) was conducted to confirm spatial separation between molecular subtypes, and Kaplan–Meier survival curves were evaluated using log-rank tests (survival package, version 3.2-11; survminer package, version 0.4.9; and TSHRC package, version 0.1–6) [38,39,40]. Significance was determined based on a *p*-value < 0.005. Hazard ratio (HR) > 1.0 indicated risk genes, whereas HR < 1.0 indicated protective genes.

### 2.3. Subtype Differential Expression and Prognostic Risk Model Construction

Differential expression analysis between the two ASIGs molecular subtypes was conducted with the limma package (version 3.48.0) [41]. Genes with *p* < 0.0001 and |log_2_FC| > 1.5 were defined as differentially expressed genes (DEGs), adjusted with the Benjamini–Hochberg false discovery rate (FDR) method (Supplementary Table S2). For prognostic model development, candidate genes were initially screened using univariate Cox regression (*p* < 0.005), followed by LASSO-Cox regression for feature selection and coefficient shrinkage.

Specifically, LASSO-Cox modeling was implemented using the glmnet R package (version 4.1-2), where 10-fold cross-validation at lambda.min identified the optimal 17-gene signature. A risk score was calculated for each patient by summing gene expression values weighted using their LASSO-Cox regression coefficients. Patients were divided into high- and low-risk groups based on the median risk score. The signature was evaluated in TCGA and externally validated in METABRIC without model refitting.

### 2.4. Independent Prognostic Evaluation and Nomogram Development

To evaluate the independent prognostic value of the ASIG-based risk score, univariate Cox regression analysis was first performed using OS as the endpoint. The variables included age, AJCC stage, N stage (i.e., regional lymph node involvement), T stage (i.e., size and local extent of the primary tumor), and the ASIG risk score. Variables with significant associations in the univariate analysis were then entered into the multivariate Cox regression model. The proportional hazards assumption for Cox regression models was assessed using Schoenfeld residuals (cox.zph). Hazard ratios (HRs), 95% confidence intervals (CIs), and *p*-values were calculated for each variable.

Independent prognostic factors (*p* < 0.05) were incorporated to construct a clinicopathologic nomogram using the “rms” R package (version 6.2-0) to estimate 1-, 3-, and 5-year OS. Model performance was evaluated using 1000 bootstrap resampled calibration curves, time-dependent receiver operating characteristic (ROC) curves, and decision curve analysis (DCA) for clinical net benefit assessment.

### 2.5. Characterization of the Tumor Microenvironment (TME)

The cellular composition and functional state of the TME were evaluated across bulk samples. The relative abundance of 22 infiltrating immune cell types was estimated using CIBERSORT (version 1.04) [42]. ImmuneScore, StromalScore, ESTIMATEScore, and TumorPurity were calculated via the ESTIMATE algorithm (R package version 1.0.13) [43]. Single-sample Gene Set Enrichment Analysis (ssGSEA) was performed (GSVA package, version 1.40.0) to quantify 28 immune cell signatures and functional pathways [44].

### 2.6. Single-Cell Transcriptomic Integration and Intercellular Communication

Preprocessed single-cell transcriptomic profiles and cluster annotations from Wu et al. (SCP1039) were adopted [36], accessible via the Broad Institute Single Cell Portal (SCP1039: https://singlecell.broadinstitute.org/single_cell/study/SCP1039, accessed on 5 March 2024). This dataset includes single-cell transcriptomic profiles derived from 26 primary pretreatment breast cancer samples.

To ensure consistency of downstream analysis within the reference dataset, standard quality filtering criteria defined in the original study were applied, including removal of cells with fewer than 200 detected genes or with >20% mitochondrial gene content.

SCISSOR (R package version 1.0.0) [45] was used to associate single-cell transcriptional profiles with the bulk-derived ASIG risk phenotype. The inputs included the TCGA bulk expression matrix, the corresponding risk phenotype, and the single-cell RNA-sequencing expression matrix. The 17 signature genes were not directly scored in individual cells. Instead, SCISSOR evaluated the similarity between single cells and bulk samples and applied penalized regression to identify cells positively or negatively associated with the bulk-level phenotype. The single-cell dataset was split by sample, and SCISSOR was independently performed for each sample using α = 0.05 and a binomial model. Cells with positive model-derived associations were classified as SCISSOR-positive, cells with negative associations were classified as SCISSOR-negative, and cells not selected by the model were considered unselected. The resulting labels were subsequently merged for downstream analyses.

Intercellular communication networks and signaling pathway flows between Scissor-defined cell populations were inferred using ligand–receptor interaction analysis implemented in the CellChat R package (version 1.1.3) [46].

All analyses were performed using published algorithms with default parameters unless otherwise specified, ensuring reproducibility of results.

### 2.7. Statistical Analysis

All computational and statistical analyses were performed using R software (version 4.1.0) and relevant Bioconductor packages (version 3.13). Statistical tests were two-sided, and *p* < 0.05 was considered statistically significant, except where specified otherwise.

## 3. Results

### 3.1. Identification of Prognostically Significant ASIG-Related Gene Subtypes in BRCA

To assess the impact of ASIGs on BRCA prognosis, we performed a univariate Cox regression on the TCGA-BRCA cohort and identified 28 ASIGs with significant prognostic value for OS (*p* < 0.005), including risk factors (e.g., APOC3, 95% CI = 1.237–2.519, HR = 1.765) and protective genes (e.g., NFKBIA, 95% CI = 0.537–0.871, HR = 0.684) (Figure 1A). Expression of these 28 genes was then subjected to unsupervised consensus clustering to identify distinct aging-associated molecular patterns. As shown by the consensus matrix for *k* = 2 (Figure 1B), the patient cohort was cleanly bifurcated into two sharp, well-defined clusters: Cluster A (*n* = 550) and Cluster B (*n* = 508). The selection of *k* = 2 as the optimal clustering solution was strictly supported by the Cumulative Distribution Function (CDF) curves, which reached an approximate plateau at *k* = 2 (Figure 1C), and the delta area plot, which demonstrated minimal relative area gain beyond *k* = 2 (Figure 1D; additional matrices for *k* = 3–6 are provided in Supplementary Figure S1). Principal component analysis (PCA) further confirmed a clear separation between the two groups, indicating distinct ASIG expression patterns (Figure 1E).

Kaplan–Meier survival analysis showed a striking difference in OS between the two clusters (log-rank *p* < 0.0001) (Figure 1F). Patients in Cluster A exhibited significantly longer survival, whereas those in Cluster B had markedly poorer outcomes. This shows that ASIG expression patterns define clinically relevant subtypes linked to distinct aging and senescence programs. Further characterization of the tumor immune microenvironment (TME) confirmed that Cluster A exhibited higher immune infiltration and ESTIMATE scores, whereas Cluster B displayed a lymphocyte-depleted, M2 macrophage-rich immunosuppressive phenotype (Supplementary Figure S4).

### 3.2. Development and Validation of a Prognostic Risk Model Based on Aging-Related Genes

**Figure 2:**
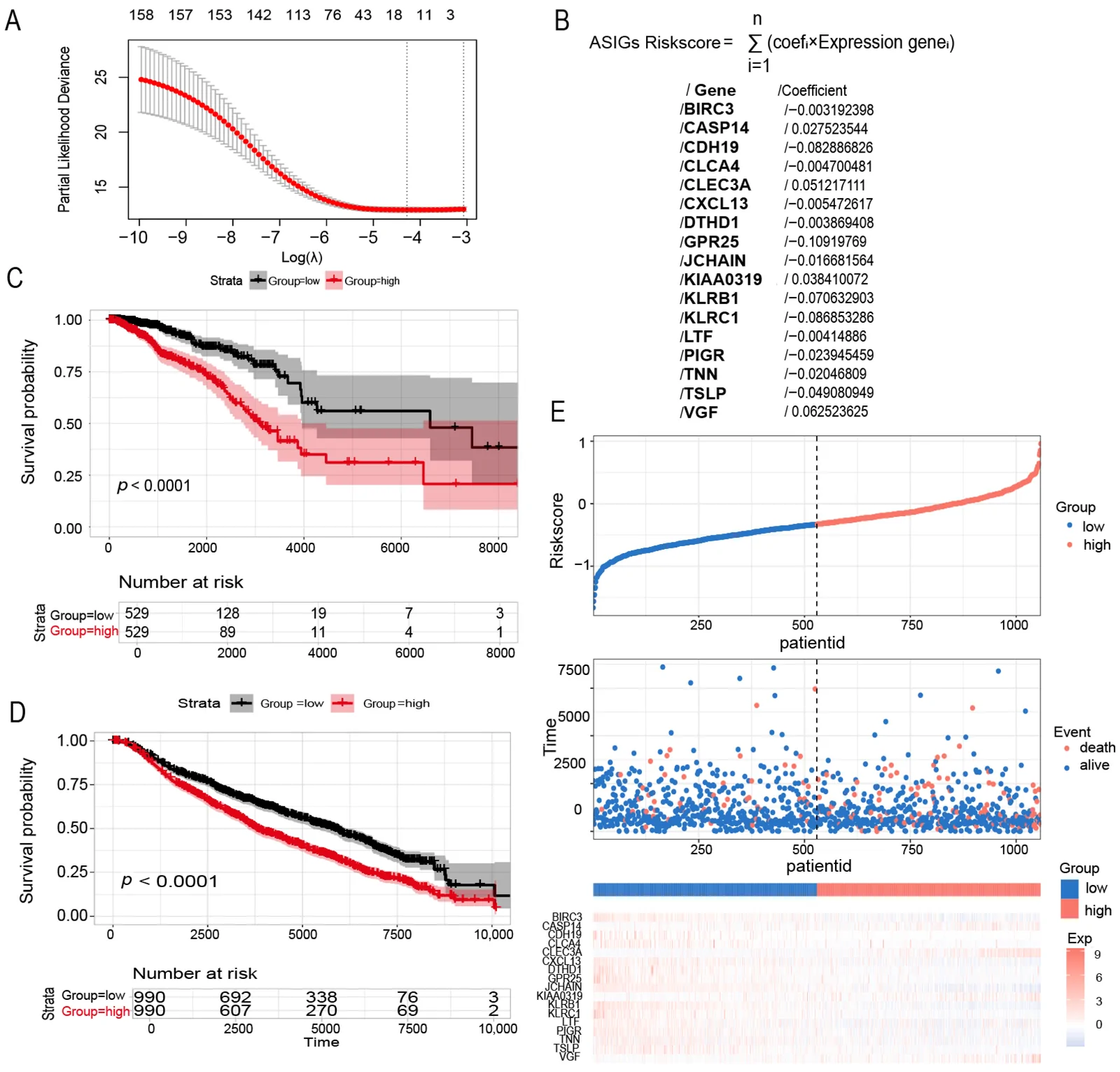
Construction and validation of the ASIG prognostic risk model. (**A**) Partial likelihood deviance versus log(λ) in the LASSO-Cox regression model. The optimal penalty parameter λ was determined using tenfold cross-validation (vertical dotted line). (**B**) The 17-gene risk score calculation formula derived from Cox regression coefficients: ASIG risk Score = (Expression of BIRC3 × (−0.003192398)) + (Expression of CASP14 × 0.027523544) + (Expression of CDH19 × (−0.082886826)) + (Expression of CLCA4 × (−0.004700481)) + (Expression of CLEC3A × 0.051217111) + (Expression of CXCL13 × (−0.005472617)) + (Expression of DTHD1 × (−0.003869408)) + (Expression of GPR25 × (−0.109197690)) + (Expression of JCHAIN × (−0.016681564)) + (Expression of KIAA0319 × 0.038410072) + (Expression of KLRB1 × (−0.070632903)) + (Expression of KLRC1 × (−0.086853286)) + (Expression of LTF × (−0.004148860)) + (Expression of PIGR × (−0.023945459)) + (Expression of TNN × (−0.020468609)) + (Expression of TSLP × (−0.049080949)) + (Expression of VGF × 0.062523625). (**C**,**D**) Kaplan–Meier curves for OS of patients in high- and low-risk groups in the TCGA cohort and (**D**) METABRIC validation cohort, evaluated using the log-rank test. Number-at-risk tables are shown below. (**E**) Risk score distribution, patient survival status (alive vs. death), and heatmap displaying signature gene expression and clinicopathological traits ranked by ascending risk score in TCGA.

To create a prognostic signature, we selected 158 DEGs between the ASIG clusters with prognostic significance (adjusted *p*  <  0.05 and |log_2_FC| >  1.5) (Supplementary Table S2). Applying LASSO-Cox regression identified an optimal 17-gene prognostic model via 10-fold cross-validation (Figure 2A). A risk score was computed for each patient as the sum of gene expression values weighted by their respective regression coefficients (Figure 2B; formula detailed in Figure 2B legend).

Patients in the TCGA cohort were divided into low-risk (*n* = 529) and high-risk (*n* = 529) groups based on the median score. High-risk patients exhibited significantly worse overall survival (Kaplan–Meier log-rank *p* < 0.0001; Figure 2C). Heatmap analyses of the signature genes revealed opposing expression profiles: protective genes with negative coefficients (e.g., BIRC3, JCHAIN, CXCL13) were enriched in the low-risk group, whereas risk-enhancing genes with positive coefficients (e.g., CASP14, CLEC3A, VGF) were upregulated in the high-risk group (Figure 2E; clinicopathological characteristics summarized in Supplementary Table S1).

To validate the model, the independent METABRIC cohort was used as an external dataset. Consistent with TCGA, patients in the high-risk group in METABRIC exhibited significantly worse OS (Figure 2D), confirming the generalizability of the prognostic risk model. The proportional hazards assumption was assessed using Schoenfeld residuals and was not violated for the high- versus low-risk comparisons in either TCGA (*p* = 0.102) or METABRIC (*p* = 0.787).

### 3.3. Development and Validation of an Aging-Related Gene Signature for Prognostic Stratification and Nomogram

**Figure 3:**
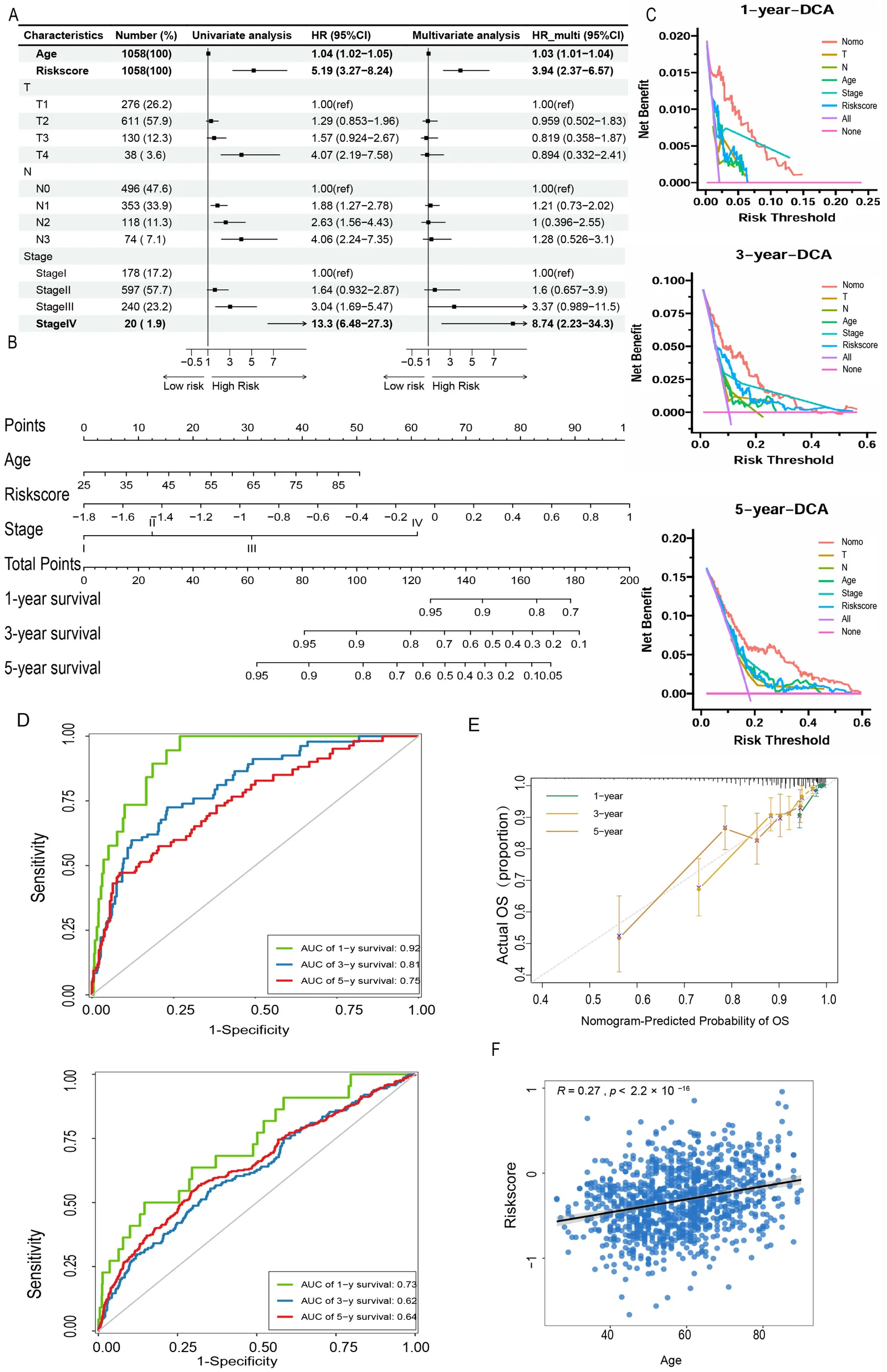
Independent prognostic value of the risk score and construction of a predictive nomogram. (**A**) Forest plots showing univariate and multivariate Cox regression analyses of clinicopathological variables and the ASIG risk score for OS in TCGA. (**B**) Clinicopathologic nomogram integrating age, AJCC stage, and the ASIG risk score to estimate the 1-, 3-, and 5-year OS probabilities. (**C**) Decision curve analysis (DCA) of the prognostic nomogram. DCA curves evaluating the clinical net benefit of the clinicopathologic nomogram (Nomo) compared with individual clinical parameters (T, N, Age, Stage, Risk score) and baseline strategies (All vs. None) for predicting 1-, 3-, and 5-year overall survival in TCGA. The nomogram consistently yields the highest net benefit across a wide range of threshold probabilities. (**D**) Time-dependent ROC curves of the nomogram in the (up) TCGA cohort and (down) METABRIC validation cohorts. (**E**) Calibration analysis of the ASIG-based prognostic model. Calibration curves comparing model-predicted and observed overall survival probabilities at 1, 3, and 5 years in the TCGA cohort. The diagonal line represents perfect agreement between predicted and observed survival probabilities. Error bars indicate the uncertainty of the Kaplan–Meier estimates. (**F**) Correlation between patient age and the model-derived risk score (*R* = 0.27, *p* < 2.2 × 10^−16^).

Univariate and multivariate Cox regression analyses were performed using OS as the primary endpoint to evaluate the independent prognostic value of the ASIG risk score (Figure 3A). In the univariate analysis, age, AJCC stage, T stage, N stage, and the ASIG risk score were all significantly associated with OS. T stage and N stage were evaluated according to the TNM classification system, reflecting the extent of primary tumor invasion and regional lymph node involvement, respectively.

Variables that were significant in the univariate analysis were subsequently incorporated into the multivariate Cox regression model. After adjustment for potential confounding factors (age, AJCC stage, T stage and N stage), the ASIG risk score remained a strong independently predictor of OS (HR = 3.94, 95% CI: 2.37–6.57, *p* < 0.001; Figure 3A) These findings suggest that the ASIG-based signature provides complementary prognostic information and may serve as a valuable tool for risk stratification in patients with breast cancer.

A clinicopathologic nomogram integrating the ASIG score, age, and AJCC stage was constructed to predict 1-, 3-, and 5-year OS (Figure 3B; Supplementary Table S1). Decision curve analysis further demonstrated that the nomogram yielded a higher net benefit than individual clinicopathological variables, as well as the treat-all and treat-none strategies, across clinically relevant threshold probabilities for predicting 1-, 3-, and 5-year overall survival (Figure 3C), supporting its potential clinical utility for individualized prognostic assessment.

Calibration curves confirmed the nomogram’s high concordance with observed OS (Figure 3C). Moreover, time-dependent receiver operating characteristic (ROC) curve analysis demonstrated favorable predictive performance, with 1-, 3-, and 5-year area under the curve (AUC) values of 0.92, 0.81, and 0.75 in the TCGA cohort (Figure 3D), and 0.73, 0.62, and 0.64 in the METABRIC validation cohort (Figure 3E), respectively. Calibration analyses were performed at 1, 3, and 5 years in both the TCGA and METABRIC cohorts (Figure 3E and Supplementary Figure S3A). In TCGA, the Brier scores of the multivariable ASIG-based model were 0.019, 0.079, and 0.124 at 1, 3, and 5 years, respectively, compared with 0.021, 0.092, and 0.145 for the reference model (Supplementary Figure S3B). In METABRIC, the corresponding Brier scores were 0.015, 0.110, and 0.200, compared with 0.015, 0.101, and 0.169 for the reference model (Supplementary Figure S3B). Thus, although the model showed relatively low apparent prediction error in TCGA, its 3- and 5-year prediction errors were higher than those of the reference model in the external METABRIC cohort, indicating attenuated long-term predictive accuracy.

To evaluate whether the ASIG risk score provides intra-subtype value, we performed survival analyses within individual PAM50 molecular subtypes (Supplementary Figure S2). High risk scores consistently stratified patients toward worse OS across most subtypes, including Luminal A, Luminal B, Basal-like, and Normal-like tumors (all *p* < 0.05; Supplementary Figure S2A–D). However, no significant separation was observed in the HER2-enriched subgroup (Supplementary Figure S1E), likely due to limited sample size (*n* = 68). Overall, these findings suggest that the risk score offers complementary prognostic insights alongside intrinsic PAM50 subtyping.

Given the aging-related nature of the signature, we next evaluated the association between the risk score and patient age in the TCGA cohort (Figure 3F). Correlation analysis demonstrated a positive relationship between age and the ASIG risk score (*R* = 0.27, *p* < 2.2 × 10^−16^). Notably, the correlation was moderate rather than strong, confirming that the risk score reflects not merely chronological aging, but rather broader tumor-intrinsic senescence- and aging-related molecular characteristics.

### 3.4. The High-Risk ASIG Signature Is Associated with an Immunosuppressive Tumor Microenvironment

**Figure 4:**
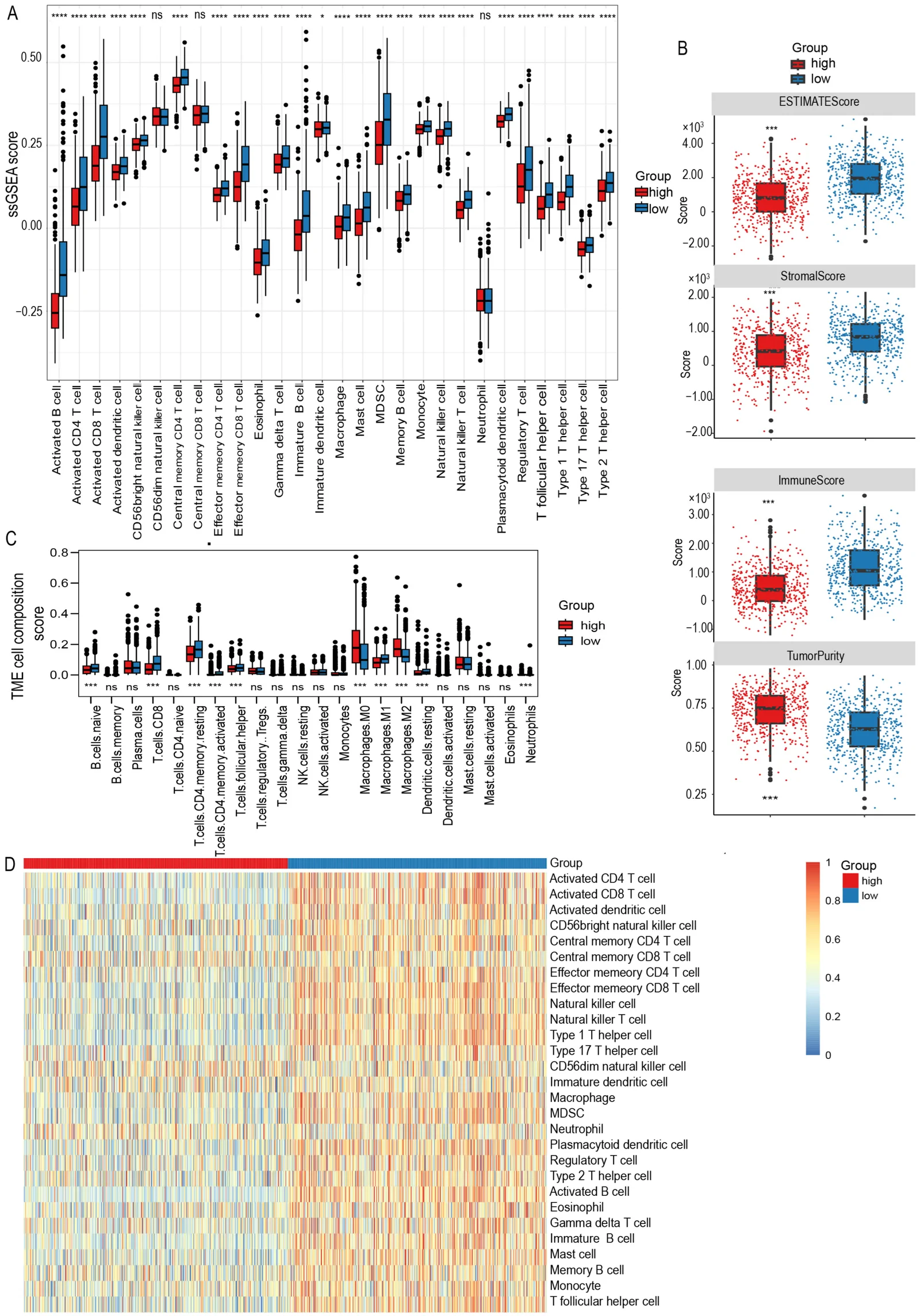
Differences in the tumor immune microenvironment between high- and low-risk groups. (**A**) Boxplots comparing the infiltration levels of 28 immune cell types in the high- and low-risk groups, as quantified using single-sample Gene Set Enrichment Analysis (ssGSEA). (**B**) Boxplots showing the differences in the ImmuneScore, StromalScore, ESTIMATEScore, and TumorPurity between the risk groups, as calculated using the ESTIMATE algorithm. (**C**) Comparison of the relative abundance of 22 immune cell subtypes in the high- and low-risk groups, estimated using the CIBERSORT algorithm. (**D**) A heatmap displaying the expression patterns of major immune cell populations across patients, who are annotated according to their assigned risk group (high vs. low). * *p* < 0.05, *** *p* < 0.001, **** *p* < 0.0001; ns, not significant (*p* > 0.05).

To delineate the biological processes underpinning the 17-gene risk score, we analyzed its associated TME landscape (Figure 4). Strikingly, the microenvironmental patterns between the high- and low-risk groups fully recapitulated the phenotypes observed in the initial ASIG molecular subtypes (Supplementary Figure S4). The high-risk group was characterized by a striking depletion of antitumor lymphocyte populations (CD4+/CD8+ T cells, activated B cells) as measured using ssGSEA (Figure 4A,D). These tumors had significantly lower ESTIMATE, Immune, and Stromal scores and higher Tumor Purity (*p* < 0.001) (Figure 4B). This lymphocyte deficit was accompanied by an enrichment of immunosuppressive M0 and M2 macrophages (Figure 4C). In contrast, the low-risk group was relatively abundant in lymphocyte populations and lower in immunosuppressive macrophages.

### 3.5. Single-Cell Transcriptomic Analysis Pinpoints Key Cellular Subsets Driving the Divergent-Prognostic TME

**Figure 5:**
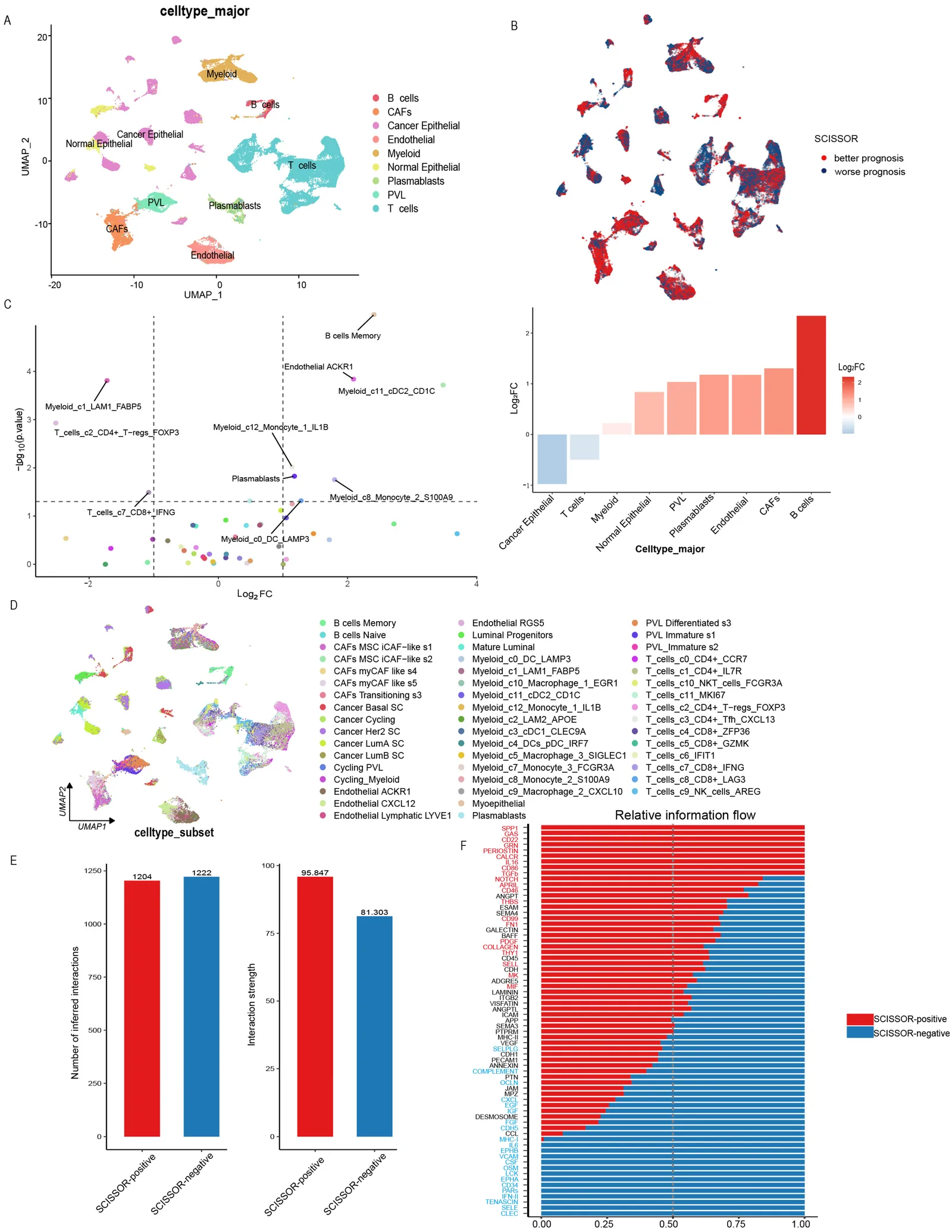
UMAP and SCISSOR-defined risk groupings highlight the heterogeneity in the BRCA microenvironment. (**A**) UMAP plot showing the distribution of 9 major cell lineages across 26 BRCA single-cell profiles. (**B**) Identification of phenotype-associated cell populations using the SCISSOR algorithm. Top: UMAP scatter plot displaying cells classified as SCISSOR-positive (associated with poor prognosis, red) and SCISSOR-negative (associated with favorable prognosis, blue). Bottom: Bar plot showing the log_2_ fold-change (log_2_FC) enrichment of SCISSOR-selected cells across the 9 major cell lineages. (**C**) Volcano plot identifying key cell subclusters driving SCISSOR-positive and SCISSOR-negative prognostic phenotypes. (**D**) Detailed distribution of cell clusters in the BRCA microenvironment, as revealed with UMAP. (**E**) Bar plots comparing the total number of inferred interactions (**left**) and the overall interaction strength (**right**) between the SCISSOR-positive (red) and SCISSOR-negative (blue) communication networks derived from CellChat. (**F**) Bar plot showing the relative information flow of major signaling pathways identified using CellChat. Red bars indicate the contribution from the high-risk (scissor-positive) cell network, while blue bars indicate the contribution from the low-risk (scissor-negative) cell network. Pathways are ranked according to their differential contribution.

We next used single-cell RNA sequencing (scRNA-seq) data from 26 primary BRCA samples to identify the cellular drivers of the high- and low-risk TME (Figure 5). Our analysis identified 9 main cell lineages (Figure 5A) and 49 fine-grained subclusters (Figure 5B). By applying the SCISSOR algorithm with default survival regression parameters (α = 0.05), we linked these single-cell profiles to the bulk risk score model to identify phenotype-associated subpopulations (Figure 5C; SCISSOR-positive cells represent poor prognosis, while SCISSOR-negative cells represent favorable prognosis). Among the 100,064 analyzed cells, 26,763 cells (26.75%) were classified as SCISSOR-positive, 24,408 cells (24.39%) were classified as SCISSOR-negative, and 48,893 cells (48.86%) were not selected by the SCISSOR model. SCISSOR-negative cells (favorable prognosis) were predominantly enriched in B cells, plasmablasts, endothelial cells, and specific myeloid lineages (Figure 5C). Specifically, enriched CD1C^+^ cDC2, endothelial ACKR1, memory B cells, and plasmablasts are the most significant contributors to the better-prognosis signature (Figure 5D), supporting efficient antigen presentation, immune cell recruitment, and sustained humoral immune responses. In contrast, SCISSOR-positive cells (poor prognosis) were characterized by an enrichment of IFNG^+^ CD8^+^ T cells, FOXP3^+^ regulatory T cells, and FABP5^+^ lipid-associated macrophages (Figure 5C,D), reflecting a dysfunctional, immunosuppressive microenvironmental state.

CellChat analysis was performed to evaluate intercellular communication networks between SCISSOR-selected cell populations (Figure 5E,F). The total number of inferred cell–cell interactions was comparable between the SCISSOR-positive and SCISSOR-negative groups (1204 versus 1222), whereas the aggregated communication strength score was numerically higher in the SCISSOR-positive group (95.8 versus 81.3) (Figure 5E). Because these values were derived from pooled cell populations without sample-level statistical testing, this difference was interpreted descriptively rather than as evidence of a statistically significant increase in overall communication strength. The flow of major signaling pathways (Figure 5F) revealed that the better-prognosis TME was significantly enriched in IFN-II, LCK, EPH, IL6, and various endothelial-related signaling pathways (including CD34, SELE, CLEC and VCAM), representing a TME constructed by active immune signaling, intact vascular function, and well-organized intercellular communication. In contrast, the poor-prognosis TME showed increased contribution of signaling pathways such as SPP1, GAS, CD22, and GRN, which drive macrophage inflammation, B cell senescence, and tumor cell migration.

## 4. Discussion

### 4.1. Prognostic Value and Clinical Independence of the ASIG Score

Breast cancer is characterized by substantial intertumoral and intratumoral heterogeneity, which continues to complicate prognostic evaluation and therapeutic decision-making. Increasing evidence suggests that, beyond chronological aging, biological aging and age-associated microenvironmental remodeling contribute substantially to tumor progression, immune dysfunction, and therapeutic resistance [10]. In the present study, we developed and validated an aging-related prognostic signature based on aging-associated genes and further integrated bulk transcriptomic analysis with single-cell transcriptomic characterization to explore its potential biological relevance. Our findings suggest that the ASIG score is associated with patient prognosis and reflects distinct immune and stromal states within the TME.

The ASIG score exhibited consistent prognostic performance across independent cohorts, including the METABRIC dataset, supporting the reproducibility and potential generalizability of the model. Notably, although age was significantly correlated with the risk score, the correlation was modest (*R* = 0.27), indicating that age accounted for only a limited proportion of the variation in the risk score. This moderate correlation demonstrates that the signature primarily captures biological senescence and microenvironmental dysregulation within the tumor rather than simply mirroring chronological age. Importantly, multivariate Cox regression analyses indicated that the ASIG score retained prognostic significance after adjustment for conventional clinicopathological variables, such as age, tumor stage [47], and molecular subtype. This independence suggests that the signature captures critical biological variations that are not fully represented by existing clinical staging or staging-related parameters, thereby offering incremental value for risk stratification.

### 4.2. Molecular Features and Biological Functions of Key Signature Genes

The biological underpinning of the ASIG score appears to be rooted in the complex interplay between tumor cells and their surrounding immune microenvironment. The signature captures an aging-associated transcriptional program in which coordinated alterations in immune regulation, epithelial integrity, and inflammatory stress collectively shape a pro-tumorigenic TME [48]. Within the model, several protective genes with negative coefficients, including KLRB1, KLRC1, and CXCL13, are closely linked to effective immune surveillance. KLRB1 and KLRC1 participate in T cell and NK cell functional regulation, and their down-regulation has been implicated in immunosenescence and impaired cytotoxic capacity [48,49]. CXCL13, a key mediator of B cell recruitment, is essential for the organization of tertiary lymphoid structures and active antitumor immunity [50]. Furthermore, multiple negatively weighted genes, such as PIGR [51], JCHAIN [52], and CLCA4 [53], are involved in epithelial barrier maintenance, mucosal immunity, or cell survival regulation. Their decreased expression reflects aging-associated epithelial dysfunction and impaired immune/epithelial interactions, which may facilitate chronic inflammation and tumor-permissive conditions within the microenvironment. The predicted downregulation of these genes in the high ASIG risk group implies a potential loss of immune functional integrity rather than immune cell depletion.

Conversely, positively weighted genes in the signature reflect senescence-associated stress adaptation, matrix remodeling, and pro-inflammatory signaling. Upregulation of CLEC3A and VGF is linked to extracellular matrix remodeling, neuroendocrine-like signaling, and cancer-associated proliferation pathways [54,55,56,57]. Notably, although CASP14 traditionally promotes terminal epithelial differentiation and barrier integrity [58], its paradoxical overexpression in high-risk tumors likely reflects perturbed epidermal-like differentiation, heightened stress adaptation, and compromised barrier integrity in aggressive tumor phenotypes. Together, the concurrent loss of protective immune-epithelial markers and upregulation of these stress-responsive risk genes mark the transition toward a SASP-rich, pro-tumorigenic TME [59].

### 4.3. Cellular Landscape and Intercellular Signaling in the TME

The bulk and single-cell transcriptomic integrative approach revealed distinct TME states that correlate with divergent prognostic outcomes [54]. Deconvolution analyses indicated that tumors with high ASIG scores exhibited reduced immune cell infiltration, particularly of cytotoxic CD8^+^ T cells—a well-established mediator of immunotherapy resistance [55]. This was accompanied by a myeloid-dominant immunosuppressive profile, characterized by the enrichment of M2 macrophages [56], FOXP3^+^ regulatory T cells (Tregs) [57], and FABP5^+^ lipid-associated macrophages [58]. Within this heterogeneous ecosystem, TAMs polarized toward the M2 phenotype serve as primary drivers of immune evasion and matrix remodeling. Upstream regulatory factors, such as Galectin-9, actively shape this immunosuppressive niche; for instance, Galectin-9 has been shown to promote cancer progression by inducing macrophage differentiation toward the M2 phenotype, leading to suppression of effector T cell responses [60]. This M2-dominant TME contributes substantially to immune checkpoint blockade (ICB) resistance. Consequently, a deeper understanding of macrophage polarization pathways and the identification of new immune targets and signaling mechanisms are essential for overcoming resistance in modern immuno-oncology [61]. Conversely, favorable outcomes correlated with enriched immune-active populations (memory B cells, plasmablasts, cDC2 dendritic cells, and ACKR1^+^ endothelial cells) that enhance antigen presentation, leukocyte trafficking, and antitumor immunity [62,63,64,65].

Cell–cell communication analyses further substantiated these distinct functional states. The low-risk TME was characterized by active IFN-II- and LCK-mediated signaling, alongside endothelial-associated pathways (CD34, VCAM, SELE, and CLEC), suggesting preserved vascular integrity and functional immune surveillance. In contrast, the high-risk TME was dominated by aging-associated immune remodeling, including SPP1-driven macrophage inflammation, B cell senescence (CD22), and growth factor activity (GRN), which collectively facilitate tumor cell migration, innate immune suppression, and disease progression [66,67,68,69,70,71].

### 4.4. Complementarity of the ASIG Score with PAM50 Subtyping and Multi-Omics Context

PAM50 is a cornerstone of molecular stratification in breast cancer [72,73]; thus, evaluating the relationship between the ASIG score and the established PAM50 intrinsic classification system is essential. Importantly, the ASIG score is not designed to replace PAM50, but rather to complement it by capturing an orthogonal biological dimension. In our analyses, the nomogram incorporating the ASIG score achieved satisfactory discriminative performance (Figure 3). More importantly, the ASIG score successfully stratified patient prognosis within specific molecular subtypes, including Luminal A, Luminal B, and Basal-like tumors. This intra-subtype stratification is highly relevant because significant clinical heterogeneity exists even among patients assigned to the same molecular subtype. For instance, our signature could potentially identify a subset of high-risk Luminal A patients who may be undertreated with standard endocrine monotherapy and might benefit from more aggressive or novel therapeutic combinations. The PAM50 subtype analyses should be interpreted with caution because the relatively small sample size of certain subtypes, particularly the HER2-enriched subgroup, together with the imbalance in risk-group distribution, may limit the statistical power of within-subtype survival analyses.

The complementarity between the ASIG score and PAM50 derives from the distinct biological dimensions they represent. While the PAM50 classification primarily reflects intrinsic epithelial features linked to cell-of-origin, proliferation, hormone receptor signaling, and HER2-related pathways [73,74], the ASIG score captures the biological age, chronic inflammatory stress, and immune remodeling of the extrinsic tumor microenvironment—a critical dimension unaddressed by intrinsic epithelial classifiers. Integrating intrinsic tumor biology with extrinsic, aging-associated TME states provides a more holistic framework for understanding breast cancer heterogeneity. Furthermore, moving beyond transcriptomic profiling toward multi-omics integration—encompassing genomic, epigenomic, and metabolomic determinants—promises to further enhance risk stratification and reveal targetable oncogenic drivers across tumor types [75].

### 4.5. Methodological Strengths and Study Limitations

This study presents several strengths, notably the development and validation of a prognostic signature rooted in the fundamental biology of aging. By shifting the focus from chronological age to the molecular hallmarks of biological aging, we provide a novel framework for breast cancer prognosis. Despite cross-platform performance shifts between sequencing and microarray cohorts, the successful validation in the large METABRIC dataset and the model’s capacity to complement PAM50 subtyping highlight its potential utility for risk stratification. Furthermore, the integration of single-cell and bulk transcriptomics offers a detailed, hypothesis-generating map that bridges molecular aging states with specific cellular dynamics in the TME. Finally, decision curve analysis (DCA) demonstrated the indicated potential clinical net benefit of the clinicopathologic nomogram across a wide range of threshold probabilities (Figure 3C).

Nevertheless, several limitations must be acknowledged. First, our findings are primarily correlational and rely on retrospective analyses of public datasets. Although the nomogram demonstrated favorable discrimination, calibration, and clinical net benefit in retrospective cohorts, its potential clinical application should be interpreted with caution. Prospective validation in independent treatment-stratified cohorts is required before the model can be used to support clinical decision-making. The model demonstrated significant prognostic separation in both TCGA and METABRIC, whereas a performance decline was observed in METABRIC (AUCs 0.73, 0.62, 0.64). This decline may partly reflect model optimism or overfitting in the TCGA training cohort. In addition, this drop also likely reflects inherent technical biases between bulk sequencing platforms (RNA-seq in TCGA vs. microarray in METABRIC), cross-cohort batch effects, and variations in cellular composition. The cohorts differed in clinical composition, treatment background, follow-up duration, and event distribution. Such differences may preserve some relative risk discrimination while reducing the accuracy of absolute survival probability estimates. Therefore, the current model should be interpreted as a preliminary prognostic framework requiring further validation and potential recalibration in additional independent and prospectively collected cohorts. Second, tests of proportional hazards via Schoenfeld residuals revealed minor deviations for certain long-term follow-up parameters [76], which may stem from late-stage sample attrition or time-varying biological hazard effects in extended cohorts [77]. Third, the functional links between the ASIG signature and mechanisms such as immune exhaustion or senescence-driven remodeling remain inferred rather than experimentally validated. Fourth, our TME characterization relies heavily on computational deconvolution algorithms. Although widely accepted, these in silico predictions are inferential and require validation using orthogonal methods, such as multiplex immunohistochemistry or spatial transcriptomics, to confirm the physical presence and spatial distribution of the inferred cell populations. Fifth, the lack of granular treatment metadata in the TCGA and METABRIC cohorts restricted our evaluation of therapy-specific responses (e.g., chemotherapy or immunotherapy); thus, the ASIG score should strictly be interpreted as a prognostic biomarker.

### 4.6. Future Perspectives and Potential Clinical Translation

These limitations highlight essential directions for future research. Experimental validation of the functional roles of the key signature genes is a critical next step. In particular, wet-lab validation—including immunohistochemistry (IHC) and immunofluorescence (IF) in independent patient cohorts, in vitro knockdown/overexpression functional assays (evaluating proliferation, invasion, and migration), and flow cytometric quantification of T cell and macrophage subsets—will be essential to confirm our computational findings. Utilizing in vitro co-culture systems and ex vivo tumor models to assess how these genes modulate immune cell activity and intercellular communication will be necessary to establish causal relationships.

To translate these findings clinically, the prognostic model must be validated in prospective patient cohorts with comprehensive annotation of therapeutic regimens. Such studies will help determine whether the ASIG score can guide therapeutic decisions, particularly in predicting response to immune checkpoint inhibitors. Additionally, integrating this transcriptomic signature with other aging hallmarks, such as epigenetic clocks [78] and telomere-associated dynamics [79], will refine our understanding of biological aging in cancer. Finally, given that cell–cell communication is highly spatially organized [80], applying spatial transcriptomics to map the localization of tumor cells expressing ASIGs relative to specific immune niches will offer deeper mechanistic insights into aging-associated tumor progression.

**Figure 6:**
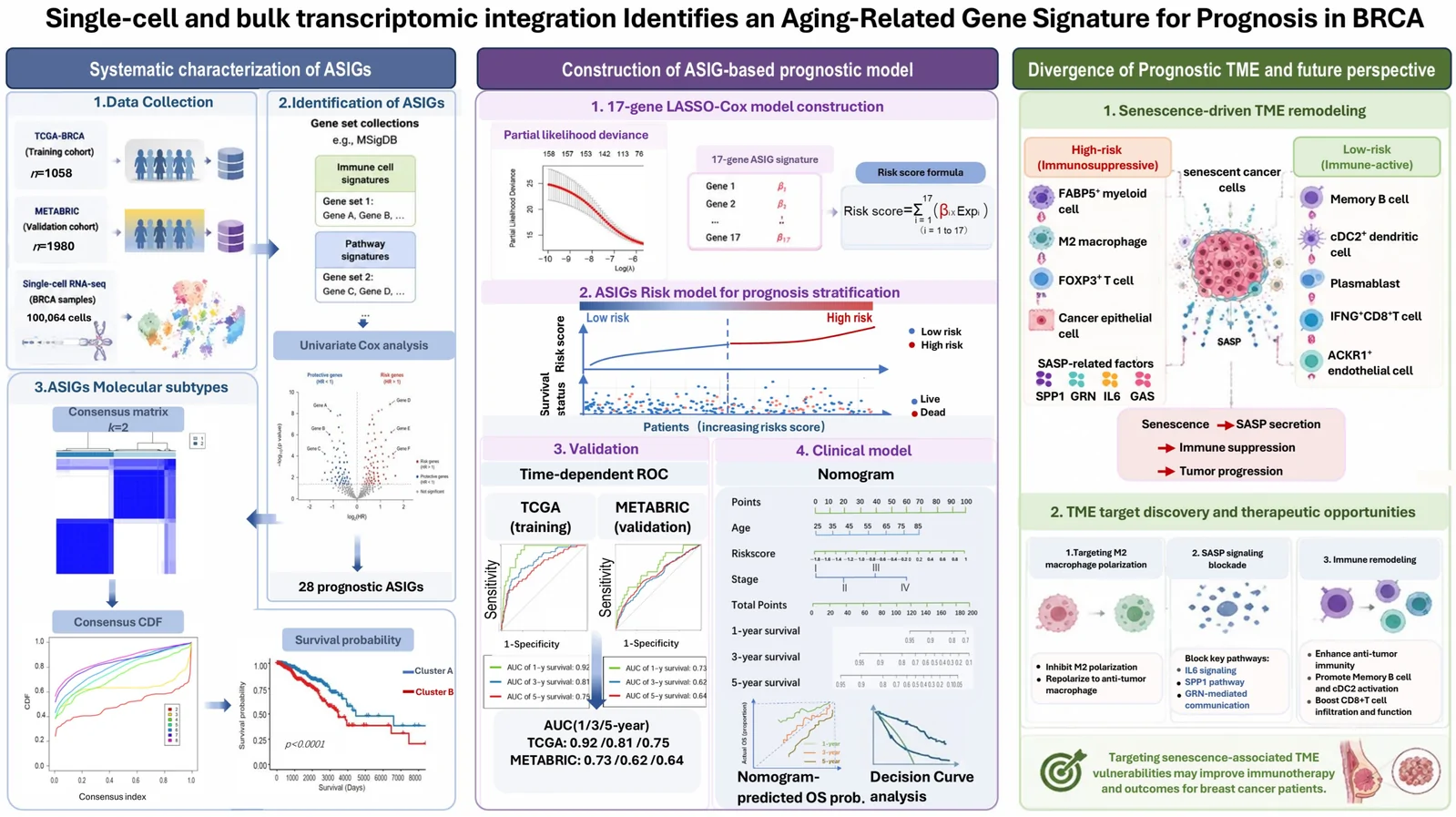
Overall workflow and mechanistic graphical model of ASIG-driven microenvironmental remodeling and prognosis in breast cancer. (**Left**) Systematic characterization of ASIGs: data acquisition across bulk (TCGA, METABRIC) and single-cell cohorts (*n* = 100,064 cells), followed by Cox regression and consensus clustering (*k* = 2) to define molecular subtypes. (**Middle**) Prognostic risk model construction and validation: development of the 17-gene LASSO-Cox signature, risk stratification, external validation, and clinicopathologic nomogram with calibration and decision curve analysis (DCA). (**Right**) Biological model of TME remodeling and future perspectives: schematic illustrating how senescent cancer cells secrete SASP factors (SPP1, GRN, IL6, GAS) to drive M2 macrophage polarization, Treg infiltration, and CD8^+^ T cell exhaustion in high-risk tumors, contrasting with the immune-active niche in low-risk tumors. Potential future strategies include targeting M2 repolarization, blocking SASP pathways, and reversing immunosuppression.

To provide a comprehensive synthesis of our findings, we integrated the computational workflow and proposed biological mechanism into a unified graphical model (Figure 6). This model encapsulates how senescence-induced transcriptional programs drive TME remodeling—transitioning from an immune-active, lymphocyte-rich niche to an SASP-mediated, immunosuppressive landscape marked by M2 macrophage polarization and T cell exhaustion—thereby determining patient prognostic divergence in breast cancer. Although the current 17-gene signature demonstrated robust prognostic performance, future studies should investigate whether a more parsimonious model can achieve comparable predictive accuracy, which may facilitate clinical implementation while maintaining model robustness.

In conclusion, our study identifies and validates a novel aging-related gene signature as a satisfactory prognostic biomarker in breast cancer, highlighting the intricate relationship between biological aging and TME dynamics. While further prospective clinical and functional validation is required, our findings suggest that quantifying the biological age of a tumor could provide a valuable dimension for risk stratification. This approach may ultimately help refine clinical decision-making, identify candidates for therapies targeting immune senescence or SASP pathways, and advance personalized management in breast cancer.

## Data Availability

Publicly available data used in this study are from TCGA and METABRIC [35]; single-cell RNA-seq data are from the breast cancer atlas at the Broad Institute Single Cell Portal (accession SCP1039, https://singlecell.broadinstitute.org/single_cell/study/SCP1039, accessed on 5 March 2024) [34]; gene set collections from MSigDB are described in the Methods. The data presented in this article are available within the text and Supplementary Materials. Further information is available from the corresponding author.

## Supplementary Materials

The following supporting information can be downloaded at: https://www.mdpi.com/article/10.3390/genes17080921/s1, Figure S1: Determination of the Optimal Number of Clusters using Consensus Clustering.; Figure S2: Prognostic stratification value of the ASIGs risk score across distinct PAM50 intrinsic molecular subtypes.; Figure S3: Evaluation of the calibration and predictive performance of the prognostic nomogram using calibration curves and Brier scores.; Figure S4: Discrepancies in the Tumor Immune Microenvironment Between ASIGs Clusters.; Table S1: Summary of clinical information from the TCGA and METABRIC.; Table S2: Differentially expressed genes (DEGs) between the two ASIG molecular subtypes.

## Abbreviations

The following abbreviations are used in this manuscript:

BRCA	Breast Cancer
scRNA-seq	Single-cell RNA Sequencing
TME	Tumor Microenvironment
ASIGs	Aging- and Senescence-Induced Genes
TCGA	The Cancer Genome Atlas
METABRIC	Molecular Taxonomy of Breast Cancer International Consortium
ssGSEA	single-sample Gene Set Enrichment Analysis
HR	Hazard Ratio
SASP	Senescence-Associated Secretory Phenotype
MSigDB	Molecular Signatures Database
GOBP	Gene Ontology Biological Process
WP	WikiPathways
TCA	Tricarboxylic Acid cycle/Citrate cycle
GEO	Gene Expression Omnibus
TPM	Transcripts Per Million
PCA	Principal Component Analysis
DEGs	Differentially Expressed Genes
LASSO	Least Absolute Shrinkage and Selection Operator
AJCC	American Joint Committee on Cancer
CDF	Cumulative Distribution Function
OS	Overall Survival
UMAP	Uniform Manifold Approximation and Projection
95% CI	95% Confidence Interval
ESTIMATE	Estimation of STromal and Immune cells in MAlignant Tumor tissues using Expression data
CIBERSORT	Cell-type Identification by Estimating Relative Subsets of RNA Transcripts
TNM	Tumor, Node, Metastasis Staging System
ADCs	Antibody–Drug Conjugates
AUC	Area Under the Curve
CAFs	Cancer-Associated Fibroblasts
CC BY	Creative Commons Attribution
cDC2	conventional Dendritic Cell type 2
DCA	Decision Curve Analysis
FDR	False Discovery Rate
ICB	Immune Checkpoint Blockade
IF	Immunofluorescence
IHC	Immunohistochemistry
IL-6	Interleukin-6
IL-8	Interleukin-8
log_2_FC	Log_2_ Fold Change
MDSC	Myeloid-Derived Suppressor Cell
NK	Natural Killer
NKT	Natural Killer T
PVL	Perivascular-like cells
ROC	Receiver Operating Characteristic
SCISSOR	Single-Cell Identification of Subpopulations with Bulk Sample Phenotypes
TAMs	Tumor-Associated Macrophages
TGF-β	Transforming Growth Factor beta
Tregs	Regulatory T cells
