# Supplementary material for: Integrated Single-Cell and Bulk RNA-Sequencing Analysis Identifies an Aging-Related Gene Signature for Prognosis in Breast Cancer
Source: Genes (Basel). 2026 Aug 4;17(8):921. doi: 10.3390/genes17080921 (PMC13512582; doi:10.3390/genes17080921)
Supplement: Supplementary file 1 [file genes-17-00921-s001.zip › genes-4422921-supplementary/supplementary Figures.pdf]

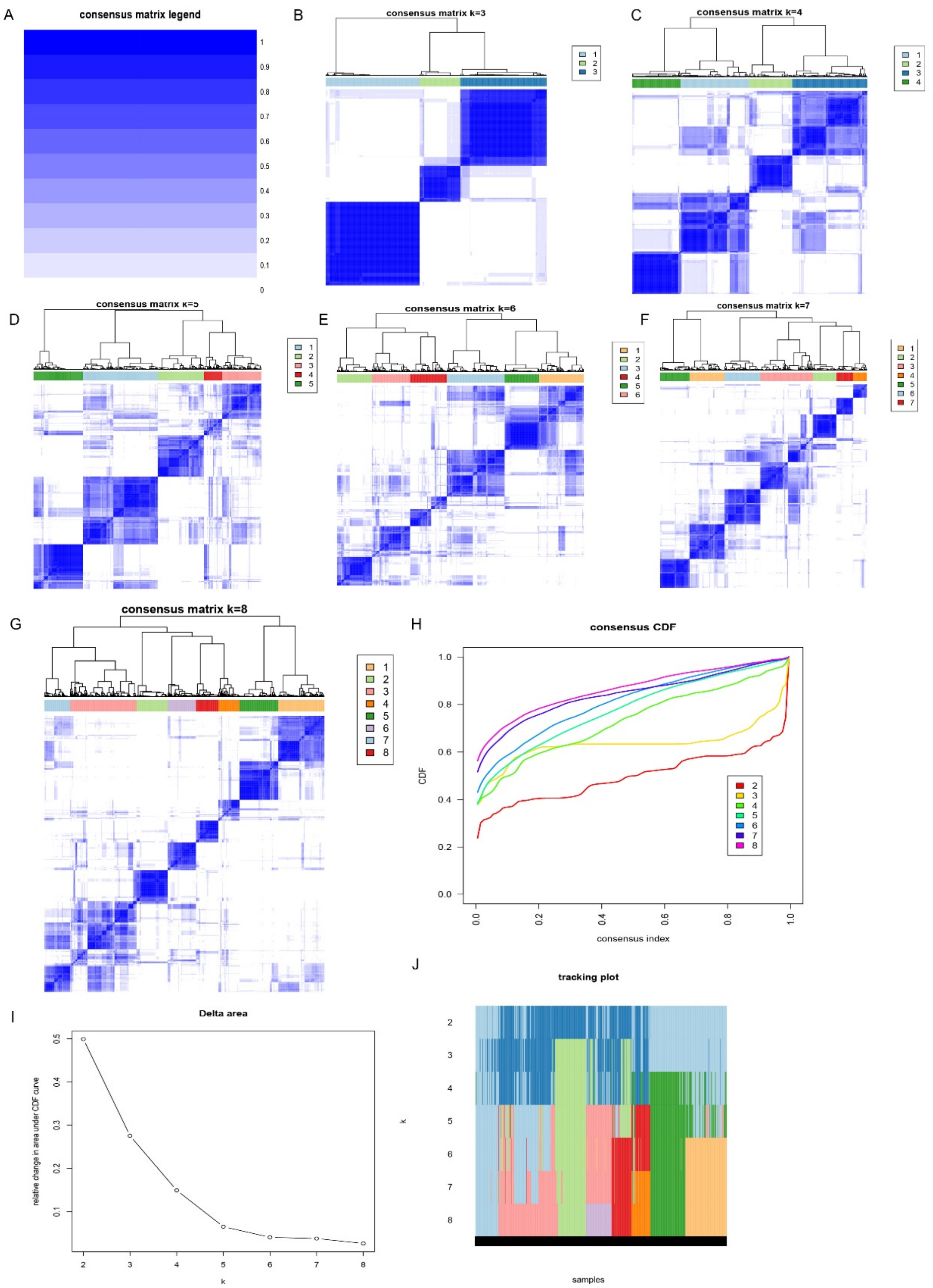

Supplementary Figure S1: Determination of the Optimal Number of Clusters using Consensus Clustering.

(A) Legend for the consensus matrix heatmap. The color gradient from white to dark blue represents the consensus index, ranging from 0 (samples never clustered together) to 1 (samples always clustered together), indicating the stability of clustering.

(B-G) Consensus matrix heatmaps for k=3 through k=8. The heatmaps visually represent the

stability of patient assignments to clusters. Sharply defined, dark blue squares along the diagonal indicate stable and robust cluster assignments.

(H) Tracking plot for k=2 to k=8, showing the cluster assignment for each sample across different values of k. This plot helps to visualize how samples are re-assigned as the number of clusters increases.

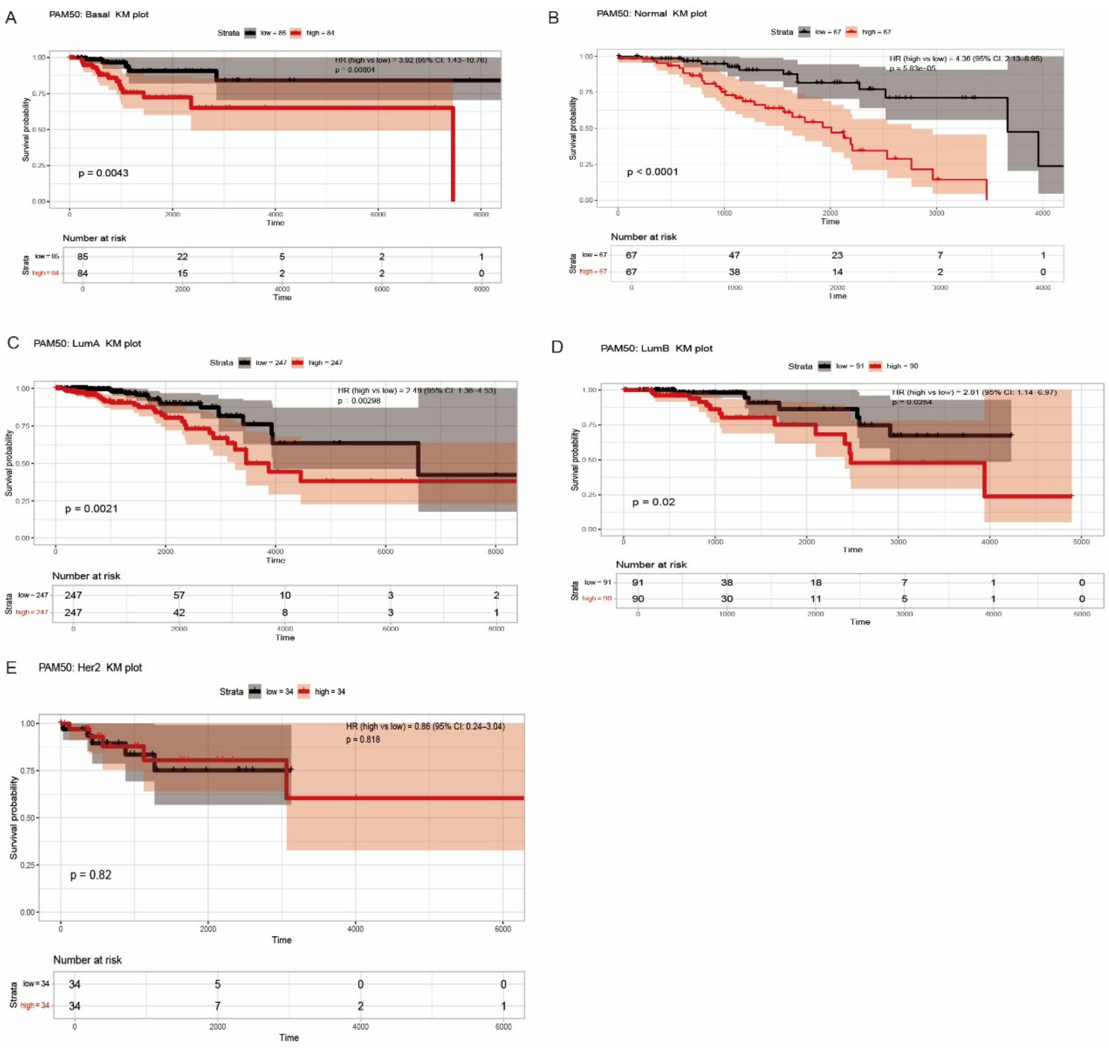

Supplementary Figure S2. Prognostic stratification value of the ASIGs risk score across distinct PAM50 intrinsic molecular subtypes.

Kaplan–Meier overall survival (OS) analyses of breast cancer patients stratified into high-risk and low-risk groups based on the median ASIGs risk score within individual PAM50 molecular subtypes:

(A) Basal-like subtype (n low = 85, n high = 84, log-rank p=0.0043; HR=3.92, 95% CI: 1.43–10.76, p=0.00804);

(B) Normal-like subtype (n low = 67, n high = 67, log-rank p<0.0001; HR=4.36,95% CI: 2.13–8.95, p=5.83×10^{-5});

(C) Luminal A (LumA) subtype (n low = 247, n high = 247, log-rank p=0.0021; HR=2.49,95% CI: 1.36–4.53, p=0.00298);

(D) Luminal B (LumB) subtype (n low = 91, n high = 90, log-rank p=0.0200; HR=2.81,95% CI: 1.14–6.97, p=0.0254);

(E) HER2-enriched (Her2) subtype (n low = 34, n high = 34, log-rank p=0.8200; HR=0.86,95% CI: 0.24–3.04, p=0.8180).

Notes: Log-rank test p -values, Cox univariate Hazard Ratios (HRs), and 95% Confidence Intervals (CIs) are annotated in each panel. Risk tables below each plot display the number of patients at risk at specific time points.

A

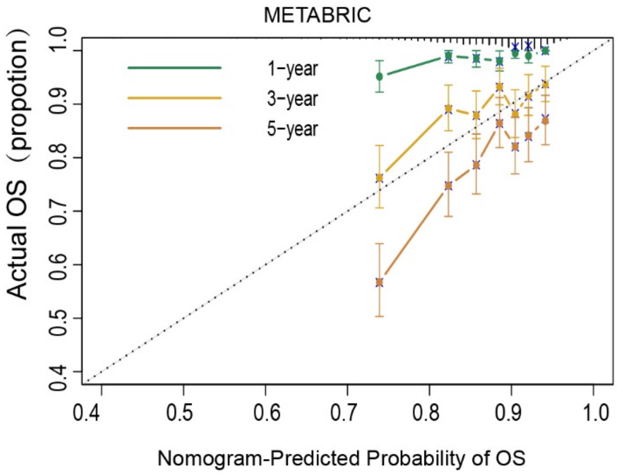

B

> Brier_compare_all

	Cohort	Time	Time_days	Reference_Brier	ASIGS_Brier
1	TCGA	1-year	365	0.021	0.019
2	TCGA	3-year	1095	0.092	0.079
3	TCGA	5-year	1825	0.145	0.124
4	METABRIC	1-year	365	0.015	0.015
5	METABRIC	3-year	1095	0.101	0.110
6	METABRIC	5-year	1825	0.169	0.200

Supplementary Figure S3. Evaluation of the calibration and predictive performance of the prognostic nomogram using calibration curves and Brier scores.

(A) Calibration curves evaluating the predictive accuracy of the nomogram for 1-, 3-, and 5-year overall survival (OS) in the METABRIC cohort. The 45-degree diagonal line represents perfect agreement between the nomogram-predicted survival probabilities and the observed

survival outcomes.

(B) Comparison of Brier scores between the reference model and the ASIGs-based nomogram for predicting 1-, 3-, and 5-year OS in the TCGA and METABRIC cohorts. Lower Brier scores indicate better overall prediction accuracy. The ASIGs-based nomogram achieved lower Brier scores than the reference model in the TCGA cohort, while comparable performance was observed for 1-year prediction and slightly higher Brier scores were observed for 3- and 5-year prediction in the METABRIC cohort.

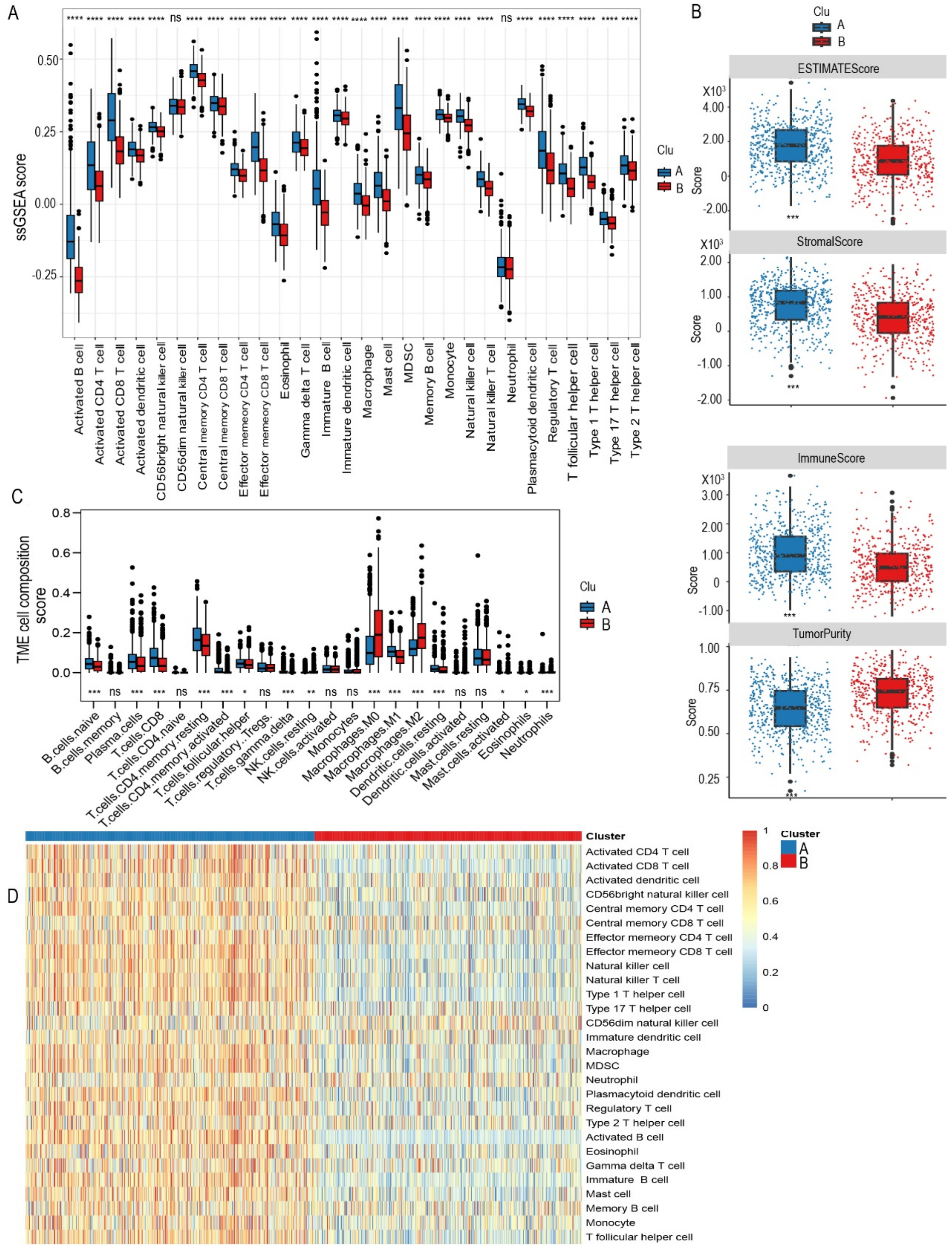

Supplementary Figure S4. Discrepancies in the Tumor Immune Microenvironment Between ASIGs Clusters.

(A) Boxplot showing the differential infiltration levels of 28 immune cell types between Cluster a and Cluster b, as quantified by single-sample Gene Set Enrichment Analysis (ssGSEA).

(B) Comparison of the ImmuneScore, StromalScore, ESTIMATEScore, and TumorPurity between the two clusters.

(C) Barplot illustrating the differential relative abundance of 22 TME cell subtypes between the two clusters, estimated by the CIBERSORT algorithm.

(D) Heatmap displaying the expression patterns of immune-related signatures, with columns representing patients annotated by cluster type (A and B).
