# Supplementary material for: Integrated Single-Cell and Bulk RNA-Sequencing Analysis Identifies an Aging-Related Gene Signature for Prognosis in Breast Cancer
Source: Genes (Basel). 2026 Aug 4;17(8):921. doi: 10.3390/genes17080921 (PMC13512582; doi:10.3390/genes17080921)
Supplement: Supplementary file 1 [file genes-17-00921-s001.zip › genes-4422921-supplementary/Supplementary.Table S1.pdf]

Supplementary Table S1 | Summary of clinical information from the TCGA and METABRIC

Characteristic	TCGA-BRCA<br>N = 1,058	METABRIC<br>N = 1,980
**Age (Median, IQR)**	58 (49–67)	62 (51–71)
**Time (mean (SD)), days**	1250.34 (1190.70)	3756.70 (2283.76)
**Clinical Stage**		
Stage I	178	501
Stage II	597	825
Stage III	240	118
Stage IV	20	10
Missing	23	526
**Tumor Stage (T)**		
T1	276	/
T2	611	/
T3	133	/
T4	38	/
Missing	3	1,980
**Node Status (N)**		
N+	554	911
N0	503	994
Missing	17	75
**Survival Status**		
Alive	911(86. 1%)	837 (42%)
Dead	147 (14%)	1,143 (58%)
**Pam50**		
Basal	169	/
Her2	68	/
LumA	494	/
LumB	181	/
Normal	134	/
NA	12	/
